# Reflections on the use of protein binders to study protein function in developmental biology

**DOI:** 10.1002/wdev.356

**Published:** 2019-07-02

**Authors:** Gustavo Aguilar, M. Alessandra Vigano, Markus Affolter, Shinya Matsuda

**Affiliations:** ^1^ Biozentrum University of Basel Basel Switzerland

**Keywords:** drosophila, GFP, guidelines, nanobody, protein binders

## Abstract

Studies in the field of developmental biology aim to unravel how a fertilized egg develops into an adult organism and how proteins and other macromolecules work together during this process. With regard to protein function, most of the developmental studies have used genetic and RNA interference approaches, combined with biochemical analyses, to reach this goal. However, there always remains much room for interpretation on how a given protein functions, because proteins work together with many other molecules in complex regulatory networks and it is not easy to reveal the function of one given protein without affecting the networks. Likewise, it has remained difficult to experimentally challenge and/or validate the proposed concepts derived from mutant analyses without tools that directly manipulate protein function in a predictable manner. Recently, synthetic tools based on protein binders such as scFvs, nanobodies, DARPins, and others have been applied in developmental biology to directly manipulate target proteins in a predicted manner. Although such tools would have a great impact in filling the gap of knowledge between mutant phenotypes and protein functions, careful investigations are required when applying functionalized protein binders to fundamental questions in developmental biology. In this review, we first summarize how protein binders have been used in the field, and then reflect on possible guidelines for applying such tools to study protein functions in developmental biology.

This article is categorized under:

Technologies > Analysis of Proteins
Establishment of Spatial and Temporal Patterns > Gradients
Invertebrate Organogenesis > Flies

## Graphical abstract

Protein Quest: Guidelines for the use of protein binders in developmental biology.

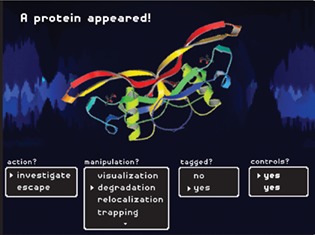

## 1 INTRODUCTION

A key question in developmental biology is how the millions of proteins expressed in each cell function in a highly coordinated manner to properly orchestrate developmental processes. Over the last few decades, genetic and reverse genetic approaches, combined with biochemical studies, have been extremely successful in gaining insight into the molecular control of development in a number of genetically amenable model systems. These studies have been largely based on characterization of mutant phenotypes by manipulation at the DNA and RNA level.

Accumulating evidence suggests that one given protein can function differently in different location, with different binding partners and different posttranslational modifications. Therefore, simple mutant analysis would not necessarily distinguish between these functions unless the mutant specifically affected one or the other of the above properties. Such mutants can be generated and analyzed, but in most cases either the wild type protein is still present, and/or the mutant is not conditional. Because of the lack of a diversified tool box to directly manipulate protein function in a more controlled manner, it has often been difficult to test models of protein functions based on mutant phenotypes.

Biological studies may suffer from such situations not only due to technical limitations but also due to lack of motivation. For example, if a model is widely accepted in the field, one may not even think of testing or challenging it. In addition, one may feel that validating a model is less rewarding than proposing a new model; in case the model would turn out to be confirmed in some of its aspect by experimental validation, reward might be minimal, although demonstrations in favor of a given model are extremely important and valuable. If a given model is widely accepted, experimental validation inconsistent with the model might be met with much skepticism. Thus, despite the accumulating number of protein‐coding genes required for developmental processes, mechanistic understanding of how proteins function together remains a difficult task.

Recently, synthetic tools based on small, high affinity protein binders such as single‐chain variable fragments (scFvs), nanobodies, Design Ankyrin Repeat Proteins (DARPins), affibodies, monobodies, and others have been applied in the field of developmental biology to directly manipulate target proteins in their complex *in vivo* settings. By fusing such binders to protein domains of well‐characterized properties (functions) and expressing these fusion proteins during development, the properties and the localization of a protein of interest (POI) can be manipulated in a predictable manner. Although these novel, synthetic tools have a great potential to fill the interpretational gap between classical genetic analyses and protein functions *in vivo*, careful controls and experimental validations are crucial to successfully apply such tools in a meaningful manner. In this review, we first summarize how synthetic protein binder fusion proteins are used and then reflect on potential guidelines for applying them to study protein function during the development of multicellular animals.

## 2 OVERVIEW OF PROTEIN BINDER APPLICATIONS IN DEVELOPMENTAL BIOLOGY

During the last decades, protein binders have emerged as a promising tool to study protein function and localization. In the following section, the main applications in developmental biology are briefly described and classified. For a more thorough description of these applications and studies in cultured cells, we refer the reader to several recent reviews covering this topic (Aguilar, Matsuda, Vigano, & Affolter, 2019; Beghein & Gettemans, 2017; Bieli et al., 2016; Harmansa & Affolter, 2018; Helma, Cardoso, Muyldermans, & Leonhardt, 2015; Kaiser, Maier, Traenkle, Emele, & Rothbauer, 2014; Pluckthun, 2015).

### 2.1 Protein visualization

**Figure 1:**
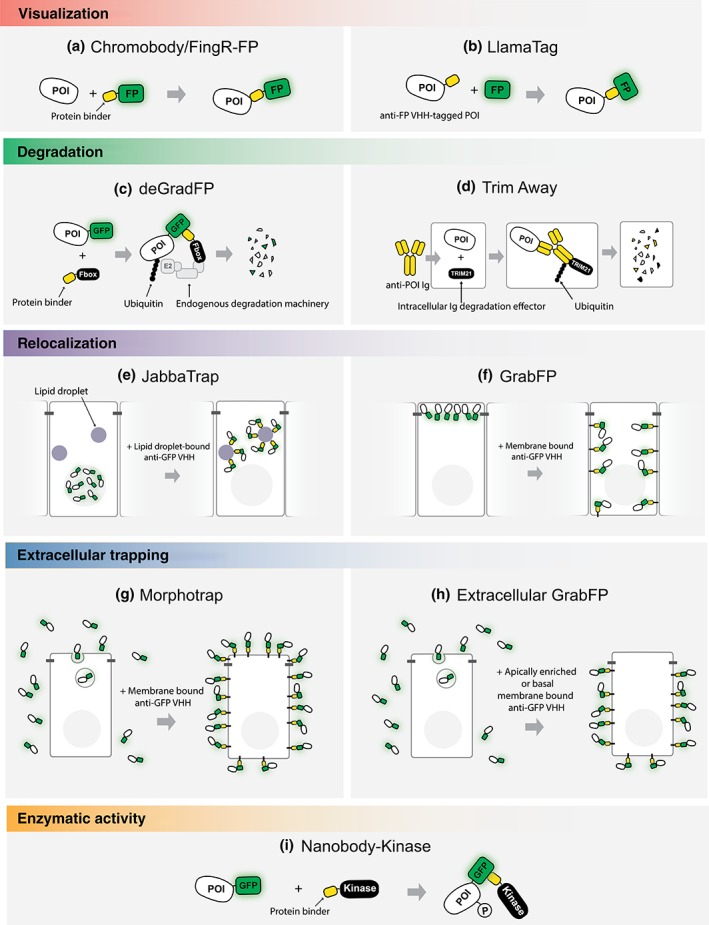
Schematic representation of the different protein binder‐based methods used in developmental biology. (a) The chromobody approach. Upon binding, protein binder‐FP fusions reveal localization of the POI. (b) LlamaTags. Upon binding to a FP, the POI fused to a protein binder recognizing a FP can be visualized via FP recruitment. (c) deGradFP. Upon binding, the GFP‐nanobody fused to an F‐box domain targets the GFP‐tagged POI for proteasomal degradation. (d) TRIM‐away. An IgG against the POI is introduced into a cell expressing TRIM21. TRIM21 sends the antibody–antigen complex to proteasomal degradation via IgG ubiquitination. (e) JabbaTrap. A GFP nanobody fused to a lipid droplet‐specific scaffold relocalizes GFP‐tagged nuclear proteins to lipid droplets to prevent their normal function. (f) GrabFP. GFP nanobodies fused to different plasma membrane scaffolds (basolateral, apically enriched or homogenously distributed) allow the relocalization of intracellular proteins in different membrane compartment. (g) Morphotrap. A GFP nanobody fused to the transmembrane domain of CD8 allows trapping of secreted molecules on the cell surface and restricting their dispersal. (h) Extracellular GrabFP. A GFP nanobody fused to different plasma membrane scaffolds permits trapping subpopulations of the secreted POI. (i) Nanobody‐kinase. A GFP nanobody fused to a kinase minimal domain induces GFP‐tagged POI phosphorylation

The direct tagging of endogenous proteins with fluorescent proteins (FPs) has been successfully employed to follow protein distribution during development. However, such tagging might be a difficult task in some organisms and the rather large size of FPs may result in functional interference. To bypass these problems, protein binders recognizing the POI have been fused to FPs. Upon binding to the POI, the FP‐protein binder allows visualization of the POI in vivo (Gross et al., 2013; Rothbauer et al., 2006) (Figure 1a).

Visualization of the POI fused to a FP might also be hampered when the half‐life of the POI is shorter than the time required for the chromophore of the FP to mature. This problem has recently been overcome by tagging the POI with a nanobody recognizing a FP (“LlamaTag”; Bothma, Norstad, Alamos, & Garcia, 2018; see Figure 1b). In the presence of uniformly expressed cytoplasmic FP, the POI fused with an anti‐FP nanobody can bind to available matured FP immediately after its translation, thereby revealing the localization of the short‐lived POI.

### 2.2 Protein degradation

Protein depletion resulting from depletion at the DNA or the RNA level, has been a widely used approach in developmental studies. However, there are some situations or factors that limit this approach, such as the slow turnover of some proteins or the masking of zygotic protein depletions due to maternal protein contribution in early embryogenesis. To accomplish acute and specific protein depletion, a GFP nanobody has been fused to an F‐box domain. Proteins containing F‐box domains determine substrate specificity of E3 ubiquitin ligases, responsible of protein polyubiquitination for proteasomal degradation. Upon binding to the GFP‐tagged POI, a GFP nanobody fused to an F‐box domain can target the POI for degradation (Caussinus, Kanca, & Affolter, 2011) (Figure 1c). Several similar approaches have been recently developed to achieve protein degradation in different model organisms (Daniel et al., 2018; Wang et al., 2017; Yamaguchi, Colak‐Champollion, & Knaut, 2019).

In many organisms, endogenous protein tagging is still difficult and time consuming. To achieve degradation of untagged proteins, Trim‐Away has been developed (Clift et al., 2017). Trim‐Away is based on the degradation of antibody‐POI complexes by TRIM21, an intracellular antibody receptor that target intracellular Ig for proteasome degradation (Mallery et al., 2010) (Figure 1d). Trim‐Away has been shown to efficiently degrade intracellular proteins in the mice oocyte and in zebrafish early embryos (Chen et al., 2019; Clift et al., 2017).

### 2.3 Protein relocalization

Proper localization of proteins is fundamental for its function. The study of protein function may require the depletion of the POI from its endogenous localization domain or its ectopic positioning to another subcellular compartment. To achieve this, a GFP nanobody has been fused to different plasma membrane scaffolding domains (GrabFP, Harmansa, Alborelli, Bieli, Caussinus, & Affolter, 2017). Upon binding to the GFP‐tagged POI, GrabFPs have been shown to relocalize the GFP‐tagged POI from the endogenous localization domain to a novel, ectopic location (Figure 1f). In a similar way, a GFP nanobody fused to a lipid droplet‐associated scaffold has been successfully employed to relocalize GFP‐tagged nuclear proteins to lipid droplets to block their function (JabbaTrap, Seller, Cho, & O'Farrell, 2019, Figure 1g).

### 2.4 Membrane trapping of extracellular proteins

The detailed dissection of the mechanism of action of paracrine factors requires interfering with specific parameters such as synthesis, release, dispersal, and degradation of the factors. To trap GFP‐tagged secreted POIs on the cell surface, a GFP nanobody has been fused to the transmembrane protein CD8 (“morphotrap”, Harmansa, Hamaratoglu, Affolter, & Caussinus, 2015). The GFP nanobody has also been fused to basolaterally localized or apically enriched scaffolds to trap subpopulations of GFP‐tagged POI extracellularly, thereby interfering with different pools of GFP‐tagged secreted POI (Harmansa et al., 2017), (Figure 1e).

### 2.5 Protein post‐translational modification

In many cases, protein function is modulated by posttranslational modifications. The function of specific post‐translational modification can be investigated by acutely and specifically regulating the modification. To achieve this, a GFP nanobody has been fused to the constitutively active kinase domain of Rho kinase. Upon binding to GFP‐tagged POI, the GFP nanobody fused to the Rho kinase phosphorylates the GFP‐tagged POI (Roubinet et al., 2017), (Figure 1h).

## 3 A GUIDELINE TO USE PROTEIN BINDERS IN DEVELOPMENTAL BIOLOGY

When deciding to use protein binders in complex cellular environments, a number of questions have to be addressed beforehand. Which binder is the best to be used? Should the POI be tagged and then manipulated via an existing and validated binder, or should binders against the POI be isolated and characterized? How should the protein binder be functionalized in order to learn more about the function of the POI and what kind of control experiments are necessary to interpret the obtained results? In the following sections, we will address these questions based on recently reported experimental data.

### 3.1 Choice of protein binders

#### 3.1.1 Protein binders against commonly used tags

For developmental biology studies in organisms in which gene editing is possible, protein binders against a tag would be the first choice, if a tag has already been inserted or can be inserted into a POI without affecting its function. A variety of protein binders derived from different protein binder platforms and recognizing a tag have been isolated and characterized (Table 1), and many of them can be easily applied without additional validations. Furthermore, given the generality of tags, protein binders against tags can be applied to a variety of POI.

**Table 1:** Summary of characterized protein binders against commonly used tags

Molecular nature	Name and antigen	Comments	Ref.
**Nanobody**	VHH4 (GFP, YFP, Venus),	Moderate increase in GFP fluorescence upon binding (Harmansa et al., 2017)	(Rothbauer et al., 2006)
	GBP2, GBP3, GBP5, GBP6 and GBP7 (GFP)		(Kirchhofer et al., 2010)
	GBP1 (GFP)	Enhances GFP fluorescence upon binding	(Kirchhofer et al., 2010)
	GBP4 (GFP)	Reduces GFP fluorescence upon binding	(Kirchhofer et al., 2010)
	Destabilized antiGFP VHH4 (GFP, YFP, Venus)	Degraded in absence of the antigen (GFP)	(Tang et al., 2016)
	Lysine‐less VHH4 (GFP, YFP, Venus)	VHH4 in which lysines have been substituted for Arginines	(Daniel et al., 2018)
	mCherry VHH		(Fridy et al., 2014)
	MoonTag (gp41, 15aa linear epitope)		(Boersma et al., 2019)
	BC2 VHH (BC2 tag, 12aa)	Not tested as intrabody.	(Braun et al., 2016)
	NbALFA (ALFAtag, 15aa)		(Götzke et al., 2019)
**Darpin**	3G86.32 Anti‐GFP DARPin (GFP)		(Brauchle et al., 2014)
	2 m22 Anti‐mCherry DARPin (mCherry)		(Brauchle et al., 2014)
	E11 and G01 Anti‐TFP DARPins (TFP)		(Vigano et al., 2018)
**scFv**	HA frankenbody (HA, 9aa linear epitope)		(Zhao et al., 2018)
	Suntag (GCN4 v4, 19aa linear epitope)	Aggregation at high levels unless fused to sfGFP‐GB1 scaffold	(Tanenbaum, Gilbert, Qi, Weissman, & Vale, 2014)
	BGP7 ScFv (BGP7, 7aa linear epitope)		(Lim, Ichinose, Shinoda, & Ueda, 2007; Wongso, Dong, Ueda, & Kitaguchi, 2017)

Among the reported protein binders recognizing a tag, nanobodies binding with high affinity and selectivity to GFP have been most widely used in the field. It has been shown that GFP nanobodies can be expressed in a functional form not only in the extracellular space but also inside cells (intrabody), which is not always the case for other protein binders. An important advantage when using GFP as a tag is that the fusion proteins can be visualized in vivo before and after manipulation using fluorescent microscopy. It should be noted that different GFP nanobodies recognize distinct, non‐overlapping domains of GFP and influence its fluorescence differently upon binding.

However, given the relatively large size, GFP may not be optimal for tagging certain proteins. Therefore, the use of recently reported intrabodies against small tags, such as SunTag (19aa), MoonTag (15aa), HAtag (9aa), and ALFAtag (15aa), promises to be a great addition to the protein binder toolbox (Boersma et al., 2019; Gotzke et al., 2019; Tanenbaum et al., 2014; Zhao et al., 2018). In most cases, the short peptide tags recognized by these intrabodies has been used in multimerized form such as to recognize single protein particles; whether these short tags also work well when integrated as a single tag remains to be tested.

In contrast to the use of GFP or other FPs as fusion partners, visualization of POIs fused to short peptide tags requires immunostaining for the tag or the POI. However, once protein binders are bound to a tag, the tag is most likely masked so that it would not be recognized by immunostaining using the anti‐tag antibody (Matsuda and Affolter, unpublished results). If good antibodies against the POI were not at hand, inserting a second tag for which validated antibodies are available would help to visualize the POI. Since intrabodies against small tags are rather new in the field, it will be critical to test whether these intrabodies can be functionalized equally well as it has been shown for GFP nanobodies.

#### 3.1.2 Protein binders against endogenous proteins

An alternative approach to the use of binders against tags is to isolate protein binders that directly recognize a POI using available screening platforms or commercial services. In this case, tagging of the POI is not required, thereby avoiding any interference arising from adding a tag. However, in contrast to well characterized protein binders against tags, the protein binders against a POI have to be first isolated and characterized in most cases. In recent years, several protein binder libraries have been published and are publicly available (see for example: McMahon et al., 2018; Moutel et al., 2016) The isolation of protein binders (nanobody, DARPins, and others) often involves the purification of properly folded proteins, since these protein binders should recognize the structure of the POI in vivo. Furthermore, several rounds of screening are usually required in order to identify a number of high affinity binders, and these binders then have to be validated before using them for in vivo applications. The specificity of protein binders may be evaluated by expressing them in the absence of the POI. So far, only a few protein binders against endogenous proteins have been used in the context of developmental biology studies.

There is an alternative route to obtain a protein binder against a POI. In case there are well‐characterized monoclonal antibodies against the POI or against specific posttranslational modifications of POI, the variable regions of the heavy and the light chains may be cloned from hybridoma lines expressing them and further engineered to generate single‐chain variable fragments (scFv). In this case, elaborated work to isolate and characterize protein binders against POI is not necessary. In case the POI functions in the intracellular milieu, the scFV has to be converted into a functional intrabody. One way to achieve this is to graft complementarity determining regions (CDRs) of a given antibody onto an scFv scaffold shown to act as an intrabody (Zhao et al., 2018).

In contrast to protein binders recognizing a tag, protein binders recognizing the POI directly interact with target POIs, and therefore may also block their functions. Indeed, in different studies, genetically encoded secreted scFvs against Pax6, Engrailed or Otx2 have been used to block these transcription factors in the extracellular milieu (Di Lullo et al., 2011; Layalle et al., 2011; Lesaffre, Joliot, Prochiantz, & Volovitch, 2007). An interesting application is to isolate protein binders specific to a posttranslational modification of POI to block the downstream event of the modification, as demonstrated by the isolation of a DARPin that selectively binds to the phosphorylated form of ERK2 (Kummer et al., 2012).

In the following sections, we will reflect on the use of different functionalizations of protein binders and the opportunities and challenges they represent for a better characterization of the function of POIs in vivo.

## 4 PROTEIN VISUALIZATION: CHROMOBODIES

In order to visualize protein distribution and dynamics in vivo, genetically encoded protein binders recognizing POIs have been fused to a FP and expressed in cells or in entire organisms to visualize the POI (Chromobodies: Panza, Maier, Schmees, Rothbauer, & Sollner, 2015; Rothbauer et al., 2006, Fingrs‐FP: Gross et al., 2013; Son et al., 2016, or Fluobodies: Nizak et al., 2003; Wongso et al., 2017, here collectively referred to as Chromobodies). Upon binding, the otherwise ubiquitously distributed chromobodies acquire the intracellular localization of the POI. This approach bypasses the generation of endogenously tagged versions of the POI (which, in some cases, is not possible due to functional interference of the tag). Moreover, this approach can reveal protein localization of newly synthesized proteins, which might not be possible by direct tagging with a FP, due to the rather slow maturation time of the FP's chromophore. Below we discuss some of the issues to be considered when using or generating novel chromobody tools.

### 4.1 Functional interference

First, chromobodies should not interfere with the function of POI upon binding (Panza et al., 2015; Son et al., 2016). One way to test this is to express the chromobodies at high levels to saturate the antigen in order to discard the possibility that small unbound proteins are rescuing the observed phenotype. It is highly recommended to do a detailed functional analysis of the POI in the presence of the binder, accompanied by biochemical protein activity assays, if available.

A possible way to avoid functional interferences by chromobody binding is the tagging of proteins with short linear epitopes and the use of protein binders against these linear peptides. An scFV against SunTag, a 19aa peptide from the yeast GCN4 transcription factor, has recently been engineered to act as an intrabody and fused to a FP to follow POIs fused to SunTag (Tanenbaum et al., 2014). So far, SunTag has been used in cell culture by using tandems of up to 32 copies of the tag in order to follow single particle dynamics. The use of such tandems dramatically increases the molecular weight of the POI after interaction with the FP‐protein binder fusions (up to 1.6 mDa with the 32x version together with FPs) and subsequently reduce protein diffusibility (Tanenbaum et al., 2014). It remains to be tested if one or a few copies of the tag are sufficient to visualize the POI.

In addition to SunTag, intrabodies against the HA‐tag (9 aa (Zhao et al., 2018)), MoonTag, a 15aa peptide from the HIV envelop protein complex subunit gp41 (Boersma et al., 2019), and ALFAtag (15aa, Götzke et al., 2019), have recently been validated. The availability of an array of intrabodies against small tags opens the exciting possibility of tagging the POI with different tags and following the dynamics of the POI via one tag at the same time as manipulating the POI via the other tag.

### 4.2 Chromobody specificity

In order to generate interpretable results, the chromobodies should specifically and exclusively bind to the POI. Ideally, the chromobodies themselves should not show any preferential distribution in the absence of the POI. Furthermore, the POI and chromobody must be co‐localized upon binding (e.g., by immunostaining of the POI) (Panza et al., 2015; Son et al., 2016). To quantify the level of POI, it might be useful to determine the background fluorescence level and subtract it from the experimental data (Boersma et al., 2019).

High chromobody expression may result in an increase of the unbound pool, making it difficult to distinguish between chromobodies that are bound to the POI and unbound chromobodies (Son et al., 2016). To distinguish between these two pools, high levels of chromobody expression should be avoided. The best way to keep low chromobody levels is to choose low expression or inducible promoters to drive chromobody expression (Panza et al., 2015). In other cases, it has been proposed to introduce a negative feedback loop by fusing the protein binder to a DNA binding domain that negatively regulates its own transcription (Gross et al., 2013; Son et al., 2016). However, these setups might result in active miss‐localization of the POI to the nucleus.

In the future, it might be interesting to use the recently developed destabilized nanobodies that are degraded unless they are bound to the antigen in order to diminish the unbound chromobody pool (Tang et al., 2016). Generating unstable versions of the other scaffolds might be important for similar reasons.

### 4.3 LlamaTags

Fusing the POI with a protein binder against a FP also permits the analysis of protein dynamics. In the LlamaTag approach (Bothma et al., 2018), a ubiquitously distributed cytoplasmic FP is recruited by a nanobody fused to the POI. This method has been used for the visualization of the dynamic distribution of transcription factors with a half‐life shorter than the time required for maturation of the GFP fluorophore (>30 min), making it impossible to visualize them by generating direct FP fusion proteins. A further advantage of this LlamaTags is that their use does not require the isolation and characterization of new binders against the POI. Although endogenous protein tagging is required, LlamaTag (~15 kDa) is smaller than GFP. LlamaTag should not affect the function of the POI, neither in the absence or presence of GFP. Since the POI does not bind to GFP directly but recruits it via the fused LlamaTag, it is unlikely that the POI is affected by interaction between the protein binder and its target.

### 4.4 Summary

- Chromobodies can visualize protein distribution in vivo in many contexts
- The use of a single copy of a short tag and chromobodies against such tags may represent a promising strategy to generate novel chromobodies

#### 4.4.1 Caveats and controls

- Chromobody expression should not perturbate the POI activity.
- Expression of the chromobodies in the absence of the POI should not show biased distribution.
- Expression level of chromobodies should be low to follow POI bound chromobodies.
- The use of multiple copies of a small tag and chromobodies against them can visualize single molecule dynamics but may disrupt the POI function by the increase in size.
- Direct binding of the chromobody to the POI may affect its function. Protein binders against small tags or LlamaTag may bypass the problem.

## 5 PROTEIN RELOCALIZATION

Recently, tools that re‐localize GFP‐tagged POIs to specific intracellular compartments in developing organisms have been reported (GrabFP, Harmansa et al., 2017; JabbaTrap, Seller et al., 2019). In both cases, the GFP nanobody has been fused to a protein scaffold with biased subcellular localization (plasma membrane, lipid droplets). These tools drag the POI away from its original compartment and relocalize it to the ectopic destination. Using these tools, the effect of removing the POI from a specific compartment and/or the effect of the relocalization of the POI to an ectopic compartment can be investigated.

### 5.1 General controls

In the first place, and as validated for the GrabFP toolkit, high levels of expression of the nanobody‐scaffold itself should not show any phenotype in the absence of a GFP‐tagged POI (Harmansa et al., 2017). In addition, and since POIs may also have strong regulation of localization, the GrabFPs can be relocalized by the POI (instead of the other way around). In this case, relocalization of the POI to the expected destination may not be possible by these methods (Harmansa et al., 2017).

So far, almost all the relocalizing tools have been generated with the VHH4 anti GFP nanobody (See Table 1). When using VHH4 anti GFP nanobody, it is important to take into account that VHH4 increases GFP fluorescence around 1.5 folds in vitro (Harmansa et al., 2017). If this increase also occurs in vivo, binding‐induced signal increase may lead to overestimation of the relocalization. The visualization of the POI by alternative methods (e.g., immunostaining) may be useful to reveal precise levels of the POI. In the future, the use of FP binders that do not change target fluorescent intensity would be ideal for GrabFPs and morphotrap approaches (see Table 1).

### 5.2 Deleterious effects of ectopic localization

If the aim of the study is to achieve a loss of function of the POI via its relocalization, the major concern is the possible deleterious effects that the relocalized POI may have in the novel destination compartment. A possible test for this would be to relocalize the POI in a background with both tagged and untagged POI (such as heterozygotes for the tagged POI). While the tagged POI would be relocalized to the ectopic compartment upon binding, the non‐tagged POI may still function properly in its endogenous location. Therefore, deleterious effects would most likely be due to the ectopic function of relocalized POIs. However, if the POI oligomerizes, non‐tagged POI can be indirectly relocalized to the ectopic compartment. It thus is indispensable to check the presence of non‐tagged POI in the original localization. The relocalization of nuclear proteins out of the nucleus has been proven a successful method (Seller et al., 2019). It is thus important to relocalize the POI to a subcellular location in which it cannot function.

Such specific manipulations are still difficult with the current tools, but a potential way to avoid the deleterious effects of ectopic localization of the POI could be to employ protein binders that disrupt the function of the POI in a specific compartment without relocalization of the POI. The generation and use of such tools might also require a fine understanding of the secretory pathways, to prevent the premature interaction and blocking of the whole pool of the POI before they interact in the compartment of interest.

### 5.3 Comparing different relocalization tools

The GrabFP toolset makes use of three different plasma membrane scaffolds. The distinct protein topology and nature of each scaffold may result in different relocalization efficiency and effects on POI function upon binding. Thus, it is difficult to directly compare the phenotypes observed between them. In the future, the generation of relocalization tools derived from the same protein scaffold with different localization signals may solve this problem.

### 5.4 Summary

- Relocalization tools can reveal the function of the POI in its specific compartments and/or the effects of the POI in ectopic compartments.
- New strategies are required for comparing the relocalization of a POI by different scaffolds.

#### 5.4.1 Caveats and controls

- When trying to achieve a POI loss of function, POI relocalization should not cause any effect in the destination compartment.
- GFP signal enhancement by VHH4 may lead to overestimation of the relocalization efficiency.
- Relocalization tools can be relocalized themselves by the POI.

## 6 PROTEIN TRAPPING

A GFP nanobody fused to the transmembrane domain of CD8 such that the nanobody is exposed to the outside of the cell (called morphotrap) has been shown to efficiently trap GFP‐tagged extracellular molecules on the cell surface (Harmansa et al., 2015) (Figure 1g). Morphotrap was originally developed to manipulate the dispersal of GFP‐Dpp/BMP, a morphogen that controls patterning and growth of the developing wing in drosophila. The morphotrap approach has recently also been applied in zebrafish and in *C. elegans* to study the role of dispersal of GFP‐tagged TGF‐β and Wnt ligands, respectively (Almuedo‐Castillo et al., 2018; Pani & Goldstein, 2018). By fusing the GFP nanobody to a transmembrane domain localized either to the apical or the basolateral domain of epithelial cells, Harmansa et al. also generated GFP nanobody fusions localized to different domains of the cell surface in the wing disc epithelium (GrabFP) (Figure 1h). These synthetic tools allow the trapping of extracellular molecules at different domains of a cell surface, either to visualize where molecules disperse or to block dispersal of extracellular molecules at different routes.

Functional analyses using the morphotrap or GrabFP approaches require that all the target molecules of the POI are tagged with GFP. In the case of Dpp, this was achieved by rescuing *dpp* mutant individuals via the overexpression of GFP‐tagged Dpp (Harmansa et al., 2015). However, given recent progress in genome engineering techniques, it would be ideal to generate GFP fusion proteins via tagging at the endogenous locus. A recent study in *C.elegans* indeed made an effort to generate endogenously tagged GFP‐Wnt and trap the latter using the morphotrap approach (Pani & Goldstein, 2018). However, GFP is relatively large as a tag (238aa) and can affect protein function. Indeed, we recently found that Dpp tagged with GFP at the endogenous locus is not homozygous viable and thus not fully functional (Matsuda and Affolter, unpublished). Thus, in such cases, equivalent trapping systems for short tags or for endogenous proteins will be required for future studies.

An alternative way to manipulate dispersal of extracellular soluble molecules is to generate membrane‐tethered forms of the molecules by genome editing. Recently, *wingless* (*wg*), which encodes a Wnt homolog in flies, has been replaced with a gene encoding a membrane‐tethered version of the protein (Alexandre, Baena‐Lopez, & Vincent, 2013). Using this approach, all the Wg molecules encoded by this modified gene are retained on the surface of the source cells. In contrast, morphotrap and GrabFP allow for a flexible manipulation of dispersal in a spatial–temporal controllable manner. For example, morphotrap and GrabFP can trap the ligand in source cells or in receiving cells; such a situation cannot be generated when using an endogenous membrane‐tethered form of the target protein. However, when comparing the results obtained via the direct membrane‐tethered approach with those obtained by the trapping approach, the efficiency of trapping molecules by morphotrap and GrabFP has to be carefully validated. In order to validate experiments using these tools, we suggest the following experiments.

**Figure 2:**
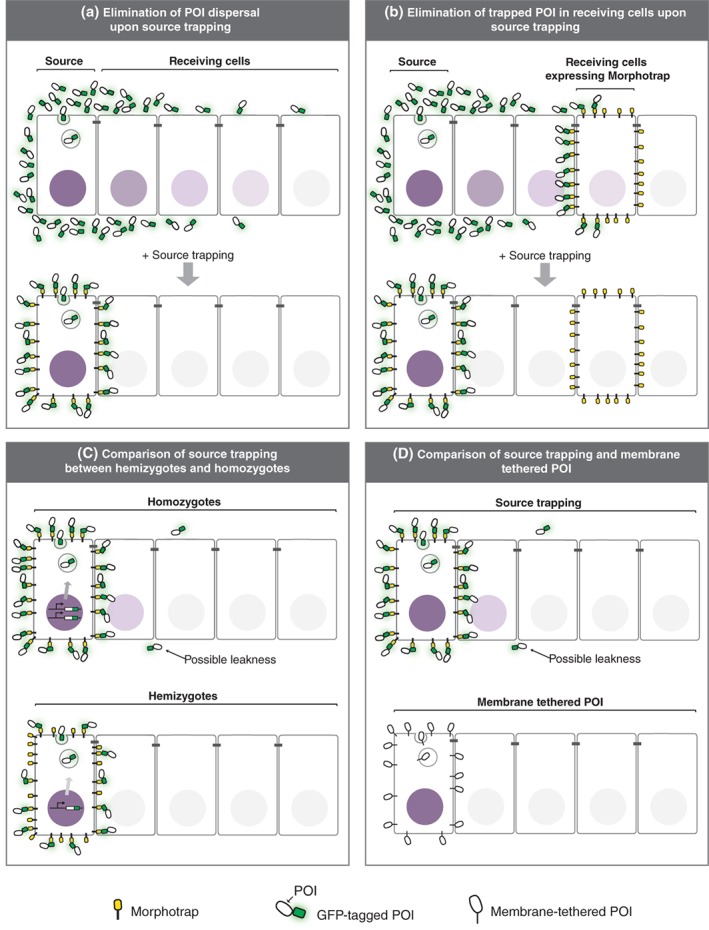
Recommended experiments to test source‐trapping of secreted ligands. (a) Trapping the POI in the source should eliminate the POI in the target tissue. If the response in the target tissue (signaling, target gene expression, etc.) is also dependent on dispersal, the response should be also eliminated. (b) POI trapped in the receiving cells should be eliminated by efficient trapping the POI in the source. (c) The source trapping phenotype in hemizygotes should not be stronger than that in homozygotes. (d) Phenotypes caused by trapping the POI should resemble those observed by the membrane‐tethered version of POI

First, when the POI is efficiently trapped in the source cells, the dispersal of the POI should be strongly affected in the receiving cells (Figure 2a). If the dispersal of the POI from the source is critical for the response in the receiving cells (signaling and target gene expression), efficient trapping of the POI in the source cells should also eliminate the response in the receiving cells. If the source and receiving cells represent separate cell lineage (e.g., are separated by a compartment boundary or are located in different tissues), the effect of blocking dispersal away from source cells is straightforward to interpret, because the receiving cells are not manipulated. However, if the source and receiving cells do not belong to separate cell lineages (or the source cells can be receiving cells), it might not be easy to separate the effects of trapping on dispersal and cell autonomous signaling.

Second, the efficient trapping of the POI at the surface of source cells should eliminate the accumulation of the target molecules by morphotrap expressed in the receiving cells at a distance from the source (see Figure 2b). In this experimental setup, even small amount of escapers are detected via the accumulation of the target molecules on the receiving cells by morphotrap or extracellular GrabFP. However, if there is more than one source contributing to the activation of the signal in the receiving cells, the POI may still be accumulated in the responding tissue if the morphotrap is very effective in one, but not in the other source.

Third, if the trapping is efficient, the phenotype caused by trapping the POI in the source in hemizygotes (with only one copy of a gene encoding the GFP fusion of the POI) should not be stronger than in homozygotes (with two copy of a gene encoding the GFP fusion of the POI), since the leakage of the POI in hemizygotes, if any, should be less than in homozygotes (Figure 2c).

However, even after performing such control experiments, one can never be certain that the trapping of the POI is 100% effective. Thus, it is desirable for certain questions to compare the phenotype resulting from the trapping of a secreted molecules with that caused by a membrane‐tethered version of POI encoded at the endogenous locus (Figure 2d).

In the case of Dpp, trapping GFP‐Dpp by morphotrap in the source resulted in undetectable extracellular GFP‐Dpp distribution, and in a severe block of Dpp signaling and downstream target gene expression in the responding target tissue. Furthermore, trapping GFP‐Dpp in the source eliminated accumulation of GFP‐Dpp by morphotrap in the receiving cells at a distance from the source. These results suggest that the trapping by morphotrap is very efficient. However, trapping GFP‐Dpp by morphotrap in the source still lead to the activation of Dpp signaling and downstream target genes at least in a single cell row in the target tissue, abutting the source cells (Harmansa et al., 2015), consistent with the possibility that trapped GFP‐Dpp in the source can still activate the signal in the adjacent, neighboring cells. However, clonal accumulation of GFP‐Dpp by morphotrap in the target tissue did not activate Dpp signal in the adjacent cells (Harmansa et al., 2015). Therefore, these results may not exclude the possibility that trapping efficiency of morphotrap is not complete and that GFP‐Dpp escaping from morphotrap in the source cells activates the signal in a single cell row in the adjacent target tissue.

In addition to testing the trapping efficiency, it is important to address the cell autonomous effects of morphotrap, extracellular GrabFP, or a membrane‐tethered version of the POI. The observed phenotype (or the lack of a phenotype) could eventually also be explained by changes in the stability of molecules or altered signaling activity rather than by blocking dispersal, especially when the source and receiving cells are not separated by lineage. For example, trapping extracellular molecules may block the dispersal of the POI but at the same time stabilize the POI on the cell surface and this may compensate for the lack of dispersal.

The differential localization of the GFP binder in morphotrap and extracellular GrabFP may allow the blocking of dispersal of extracellular molecules on different routes (Harmansa et al., 2017). However, as mentioned in the protein relocalization section, comparison of the phenotypes caused by morphotrap with those caused by extracellular GrabFP requires careful analyses. Although morphotrap and GrabFPs are based on the same GFP nanobody, they are fused with different transmembrane scaffolds. Therefore, the binding efficiency can be different depending on the expression level and topology of the GFP nanobody on the cell surface (as already mentioned in the protein relocalization section). Furthermore, the stability and effects on signaling can be different between different tools. For example, trapping the POI on the cell surface of the target tissue may show a stronger effect than trapping the POI only on the basolateral surface of the target tissue. This result may suggest the presence of an apical fraction of POI critical for a given developmental process. However, the different phenotype could also be explained if morphotrap blocks signaling stronger than the basolateral trap or if the trapping efficiency of the basolateral trap is simply lower than that of morphotrap. Therefore, differences in phenotype resulting from different tools may not reflect the differential localization of molecules.

### 6.1 Summary

- Morphotrap can efficiently trap GFP‐tagged POI and block their dispersal.
- Generation of trapping systems using short peptide tags and their respective binders may be critical in the future.

#### 6.1.1 Caveats and controls

- Efficient trapping of the POI in the source should eliminate the POI in the neighboring receiving cells, and if the dispersal of the POI is critical, the response in the receiving cells.
- Efficient trapping of the POI in the source should eliminate POI accumulation by morphotrap in the neighboring receiving cells.
- Upon efficient trapping the phenotype in hemizygotes should not be enhanced when compared with that in homozygotes.
- If possible, compare the trapping phenotype of the POI with a membrane‐tethered version of the POI.
- Cell autonomous effects by morphotrap should be carefully analyzed.
- Endogenous GFP tagging is ideal but may not work in all cases.

## 7 PROTEIN DEGRADATION

Different methods that allow to degrade proteins during the development of multicellular organisms have recently been reported, including deGradFP, zGrad, Trim‐Away, and auxin‐based degron methods (Caussinus et al., 2011; Clift et al., 2017; Daniel et al., 2018; Yamaguchi et al., 2019). These methods aim to rapidly and acutely degrade target proteins in order to investigate protein functions. However, each method has advantages and disadvantages.

### 7.1 DeGradFP

Degradation of GFP‐tagged proteins via the expression of a GFP nanobody fused to a F‐box is a powerful tool to investigate protein function. While the deGradFP approach has originally been reported to be effective in Drosophila, similar methods have in the meantime been reported in *C. elegans* (Wang et al., 2017), zebrafish (Daniel et al., 2018; Shin et al., 2015; Yamaguchi et al., 2019) and in plants (Baudisch, Pfort, Sorge, & Conrad, 2018).

Since large numbers of endogenous GFP protein trap lines are available in model system such as *C. elegans*, flies and mice, deGradFP and similar approaches can relatively easily be applied to many proteins (Nagarkar‐Jaiswal et al., 2015; Nagarkar‐Jaiswal, Manivannan, Zuo, & Bellen, 2017). However, since these resources are based on random insertion of transposons that hijacks endogenous splicing events, non‐tagged versions of the POI might still be generated by alternative splicing and complicate the functional analyses (or make the analyses impossible) (Kanca, Bellen, & Schnorrer, 2017). Thus, to better study protein function via protein degradation, tagging of the entire pool of a POI is essential. Furthermore, it has also been shown that not all proteins can be degraded by deGradFP (Caussinus et al., 2011). Since ubiquitination is dependent on the target protein, degradation efficiency is likely to be influenced by the topology of the F‐box in deGradFP and by the sequence of the POI. Therefore, despite its potential, deGradFP and similar methods do not represent general methods to degrade any POI. In the future, one way to expand the deGradFP type approach may be to generate protein degradation tools using a battery of intrabodies against short peptide tags (SunTag, HAtag, MoonTag, etc.).

### 7.2 Trim‐Away

In contrast to protein degradation methods that require intensive genome engineering to introduce a tag, Trim‐Away does not require any modification of the target protein. Trim‐Away degrades endogenous proteins in mammalian cells via the injection of an antibody and Trim21, an intracellular antibody effector which targets the antibody for degradation. Because protein degradation is dependent on Trim21‐mediated antibody degradation but not on ubiquitination of the POI, the efficiency of the degradation should not dependent on the target proteins, unlike in the deGradFP approach. Although Trim21 is expressed in many cell types (Clift et al., 2017), strong POI depletion may rely on overexpression of Trim21 or injection of recombinant Trim21. Because Trim21 recognizes the Fc region of the antibody, the method can only be implemented using the full‐length form of the immunoglobulins (Ig). The big size of the Igs may thus hamper their use to degrade the less accessible proteins (such as nuclear proteins), however, this can be overcome by using nanobody‐Fc fusions, which is smaller than the full length form of Ig (Clift et al., 2017).

These features make Trim‐Away easily and generally applicable in mammalian cells as long as delivery of antibody and Trim21 to target cells is possible. Recently, the method was successfully applied in zebrafish embryos by injection of Trim21 and the antibody into the embryo yolk (Chen et al., 2019). The most critical point to consider when applying Trim‐Away is to use a well‐defined and specific antibody in order to avoid any off‐target effects.

For the detail experimental protocol on the use of Trim‐Away, we refer the readers to the recently published protocol (Clift, So, McEwan, James, & Schuh, 2018).

### 7.3 Summary

- A variety of protein degradation tools are now available in several model organisms.
- Protein degradation tools using small tags remain to be developed and tested in the future.

#### 7.3.1 Caveats and controls

- For the deGradFP approach, GFP‐tagging of the entire pool of the POI is critical.
- For Trim‐Away, prior manipulation of the POI is not necessary, but the quality of monoclonal antibody is important.

## 8 ENZYMATIC ACTIVITY

An interesting strategy to manipulate protein function is the use of protein binders to confer substrate specificity to enzymatic domains. Along these lines, the GFP nanobody has been fused to the minimal kinase domain of Rho kinase. Upon binding, the synthetic kinase induced phosphorylation of a GFP‐tagged target protein (Roubinet et al., 2017). The aim was to study the behavior of asymmetrically dividing neural stem cells when activated myosin was increased in the apical cortex. Since the phosphomimetic myosin forms are not capable to efficiently recapitulate protein activation (Vasquez, Heissler, Billington, Sellers, & Martin, 2016), this nanobody‐mediated phosphorylation can be of major interest when studying the sufficiency of phosphorylation in this and other contexts.

The first step of tool validation in this scenario is to check that the POI is post‐translationally modified upon expression of the fusion construct. This can be achieved by employing sensors of protein activation or antibodies that recognize only the modified POI.

In a second step, it is fundamental to check for off‐target effects and to verify that no phenotypes are induced upon expression of the enzymatic domain alone. Accordingly, Roubinet and colleagues did not observe a phenotype upon expression of the Rho kinase only. However, tool validation will require a thoughtful analysis that could include, for example, the analyses of possible changes in the global phosphoprotein profiles using mass‐spectrometry.

In their analyses, the authors further functionalized the nanobody‐kinase fusion by adding an apical scaffolding domain. As predicted, the expression of this fusion protein resulted in the accumulation of phosphorylated POI in the apical cortex. It would have been very interesting to distinguish whether the accumulation of phosphorylated myosin was due to kinase activity or apical trapping of myosin. To discriminate between these possibilities, the use of a nanobody fused to the apical localization domain alone or the use of the same construct with a “dead” kinase domain, would have been a valuable tool to test whether myosin relocalization was by itself sufficient to accumulate the phosphorylated form. Supporting this hypothesis, cortical relocalization of Myosin‐GFP has been shown to be sufficient to localize myosin activity (Harmansa et al., 2017; Pham et al., 2018).

### 8.1 Summary

- The synthetic kinase may phosphorylate the POI in a predicted manner.
- Manipulation of enzymatic activity via protein binders remains to be explored.

#### 8.1.1 Caveats and controls

- The expression of the enzymatic domains alone should not cause any effect. Off‐target enzymatic activity must be analyzed.

## 9 CONCLUSSION

Protein binders have emerged as promising tools to manipulate protein functions in a predictable manner and over the past few years, a variety of protein binders and their applications have been generated and used to address questions in the field of developmental biology. However, as new tools are being generated, understanding the pros and cons of the tools and the proper implementation of controls is important in order to interpret the obtained results. We believe that this review can serve as a guideline for developmental biologists to better design and interpret experiments using protein binders.

## CONFLICT OF INTEREST

The authors have declared no conflicts of interest for this article.

## RELATED WIREs ARTICLE

https://doi.org/10.1002/wdev.214
